# Guidelines for the Prevention and Treatment of Opportunistic Infections in HIV-Exposed and HIV-Infected Children Now Online

**Published:** 2013-11-08

The *Guidelines for the Prevention and Treatment of Opportunistic Infections in HIV-Exposed and HIV-Infected Children* are now available on the AIDSinfo website ( http://aidsinfo.nih.gov/contentfiles/lvguidelines/oi_guidelines_pediatrics.pdf ). These guidelines update the last version of the guidelines published in 2009. They are intended for use by clinicians and health care workers providing medical care for human immunodeficiency virus (HIV)-exposed and HIV-infected children in the United States.

The guidelines include a discussion of opportunistic pathogens that occur in the United States and ones that might be acquired during international travel, such as malaria. The section for each opportunistic infection (OI) includes a brief description of the epidemiology, clinical presentation, and diagnosis of the OI in children; prevention of exposure; prevention of first episode of disease; discontinuation of primary prophylaxis after immune reconstitution; treatment of disease; monitoring for adverse effects during treatment, including immune reconstitution inflammatory syndrome (IRIS); management of treatment failure; prevention of disease recurrence; and discontinuation of secondary prophylaxis after immune reconstitution. Recommendations are rated using a system that indicates the strength of each recommendation and the quality of evidence supporting it.

Major changes in the guidelines include 1) greater emphasis on the importance of antiretroviral therapy (ART) for preventing and treating OIs, especially those OIs for which no specific therapy exists; 2) increased information about the diagnosis and management of IRIS; 3) additional information about managing ART in children with OIs, including potential drug–drug interactions; 4) updated immunization recommendations for HIV-exposed and HIV-infected children, including pneumococcal, human papillomavirus, meningococcal, and rotavirus vaccines; 5) addition of sections on influenza, giardiasis, and isosporiasis; 6) elimination of sections on aspergillosis, bartonellosis, and human herpes virus (HHV-6 and HHV-7) infections; and 7) updated recommendations on discontinuation of OI prophylaxis after immune reconstitution in children.

